# Content validity of the Demand and Ability Protocol – A dialogue tool involving stakeholders in exploring demands and abilities at work

**DOI:** 10.1177/10519815241300408

**Published:** 2025-01-21

**Authors:** Marie-Louise Pauhlson, Teresia Nyman, Magnus Svartengren, Kristina Eliasson, Margareta Torgén, Sofia Paulsson, Therese Hellman

**Affiliations:** 1 Department of Medical Sciences, Occupational and Environmental Medicine, Uppsala University, SE-751 85 Uppsala, Sweden; 2 Department of Occupational and Environmental Medicine, Uppsala University Hospital, SE-751 85 Uppsala, Sweden

**Keywords:** work ability, ICF, dialogue tool, content validity, return to work, think aloud interview, workplace adaptations

## Abstract

**Background:**

The ICF-based Demand and Ability Protocol (DAP) is a dialogue tool to be used when assessing the balance between an employee's functional ability and the demands of their work and as a supportive measure for discussing work ability, workplace adaptations and return to work. The DAP has been used in Sweden since the twenty-first century, however revision nor content validity has not yet been evaluated.

**Objective:**

To update and revise the Demand and Ability Protocol and to assess the content validity of the dialogue tool.

**Methods:**

A qualitative design was used involving a multidisciplinary expert panel (n = 7) and a group of experienced DAP-users (n = 13) who participated in think-aloud interviews. Data were analyzed with thematic analysis.

**Results:**

Several significant changes were made to the domains to enhance the clarity and conciseness of the DAP. Some changes in formulations were identified and changed, even though the questions were mostly perceived as clear and relevant. Overall, the DAP can be considered as a dialogue tool with good content validity.

**Conclusion:**

The revision and validation process of the DAP have clarified the theoretical concepts and their link to the ICF, establishing a strong basis for the dialogue tool's content.

## Introduction

Work ability is crucial for active participation in the workforce and can be defined as the combination of health, functioning, basic capacity, standard competence, and relevant occupational abilities concerning work tasks and the work environment.^1–3^ The definition of work ability thus addresses an individual's potential to perform work tasks while considering personal health, working conditions, mental resources, and the combination of both the individual and the situation.^4–6^ Adaptations in the workplace to support employees with reduced work ability and the possibilities to moderate them, whether in pre-rehabilitation, in the event of declining work ability, or in the case of sick leave or decreased productivity, have been evaluated as crucial factors for being able to work.^6,7^

The employer's obligations under the EU Occupational Health and Safety (EU-OHS) legislative are pivotal for achieving sustainability in the working conditions for all employees.^8^ In Sweden, employers have a continuous responsibility to regularly assess the need for work adjustments for employees and to involve the concerned employee in the examination, design, and follow-up of these work adjustments.^9^ However, it can be a challenge for employers to translate laws and regulations into everyday workplace practices and to move from word to deed.^7,10,11^ Research indicates that using a structured dialogue tool during workplace-oriented three-party meetings involving the employer and the employee, facilitated by a healthcare representative, can be effective in addressing decreasing work ability.^12–15^

Different dialogue tools have been developed to support individuals returning to work after sickness absence. These include the Workplace Dialogue for Return to Work,^16^ the Problem-Solving-based Intervention,^17^ and the Tool for Supporting a Gradual Return To Work.^18,19^ These tools have been developed for individuals on sick leave due to common mental disorders, stress-induced exhaustion disorder, and musculoskeletal disorders. They comprise interview models, including various convergence dialogue meetings. Studies have shown these dialogue tools to promote and enhance the return to work (RTW) process,^18–22^ which, by extension, can have an impact in reducing the duration of illness-related absences.^23^

Another dialogue tool is the Demand and Ability Protocol (DAP).^24^ The DAP is generic and can be used regardless of diagnoses and work situations. The only prerequisite is that the individual has an ongoing employment. It can be used in the case of a decreasing ability to work, if repeated short-term absences are at hand, as well as in the case of illness and disability with or without sick leave. Research has shown that the DAP's clear structure is appreciated by those using it^25,26^ and that it is a supportive measure for discussing work ability and RTW. Additionally, it can help the employer and employee in concrete planning of workplace interventions and adaptations.^25,26^

The DAP is linked to The International Classification of Functioning, Disability and Health (ICF) to enable a common language among stakeholders involved in the dialogue process.^27,28^ The ICF covers aspects of social, body, and participatory functions which interact with the current context providing an extensive framework of abilities relevant for discussing work ability and RTW.^28^

Nonetheless, the content of the DAP has not yet been updated since it first was introduced in Sweden at the beginning of the twenty-first century, nor has its content validity been evaluated. Content validity refers to the extent to which an assessment instrument or tool covers the intended content domain or the construct it is intended to measure. In this case, it assesses how well the DAP addresses work ability and if its formulations are suitable and easily understandable.^29^ This information is essential to provide evidence of the DAP being relevant for assessment purposes and representative of the targeted construct.

Thus, the aim of the study is to update and revise the content of the ICF-based instrument Demand and Ability Protocol (DAP), with the consensus of a research group, an expert panel, and health professionals experienced in using the DAP. Additionally, the study aims to assess the content validity of the dialogue tool.

## Method

This qualitative study^30^ is based on accumulated knowledge from the research group, a multidisciplinary expert panel, and experienced DAP-users in order to update, revise, and assess the content validity of the DAP.

### The Demand and Ability protocol

The DAP is based on the Functional and Ability List, a checklist originally developed in the Netherlands where it was used as an insurance medical instrument in assessing disability pension.^24^ It was further developed in Norway within the area of occupational health as a dialogue tool for assessing the balance between an employee's functional ability and the demands of their work.^24^ No copyright is registered for the DAP and it has been used in its current edition in various clinical settings in Sweden since the beginning of the twenty-first century. It has been translated into Swedish in close collaboration between the developers of the DAP in Norway and specialists in occupational medicine in Sweden, among them one of the co-authors.

The structured DAP dialogue is held in a three-party meeting including the employee, the employer, and a facilitator with medical competence from healthcare or occupational healthcare, trained in the use of the dialogue tool. The DAP generates information that should be included in the medical record, as some items within the dialogue tool can call for answers that necessitate medical assessment and judgment.

The original version of the DAP consists of six domains: 1) psychological and cognitive ability, 2) basic skills and social ability, 3) tolerance for physical conditions, 4) ability to work dynamically, 5) ability to work statically, and 6) ability to work certain hours. Each domain comprises items where both the work demands and the employee's functional ability are rated on a three-point scale by the employee and the employer, in collaboration. With guidance from the facilitator, the DAP is conducted systematically with the aim of identifying measures and adaptations at the workplace. It is a prerequisite for the facilitator to have some knowledge of the patient's work requirements and functional ability to explain and adjust the items accordingly to fit the current situation for both the employee and the employer. The dialogue results in a summary of which functions do not meet prevailing demands, as well as functions that are rated higher than what the work demands. Any imbalances identified are then discussed regarding what can be done to balance the job demands with the individual's level of ability. This is concluded in a plan for the actions and adaptations agreed upon for the employee, ensuring clarity and ease of follow up. The summary of the planned actions is signed by both the employee and the employer, with each receiving a copy to take home for future updates and revisions.

### Content validity procedure

*Review and construction of the first revised version.* Based on previous research and the ICF, the original version of the DAP was revised, and a preliminary version of the updated DAP was developed by the research group in collaboration with two of the developers of the Norwegian version of the DAP.

*Review of the first revised version.* A brief survey was distributed to a multidisciplinary expert panel to investigate the revised content of the preliminary version of the DAP. The aim was to ascertain whether today's requirements in working life were captured in the current domains and items, identify if something was missing, outdated, or could be removed, inspired by Davis et al.^31^ Furthermore, the formulations and wordings were evaluated.

*Review and construction of a pre-final version.* The opinions gathered from the expert panel were reviewed by the research group. Based on these results, a pre-final version of the DAP was constructed.

*Review of a pre-final version.* Health professionals who were experienced in using the DAP were interviewed with the aim of assessing the content validity of the domains and items in the pre-final revised DAP. The assessment focused on representativeness, relevance, clarity, and wording, inspired by Lecours et al.^32^

*Review and construction of the final version.* Based on all input received from both the expert panel and experienced users, the research group developed a final version of the updated and revised DAP.

**Figure 1.**
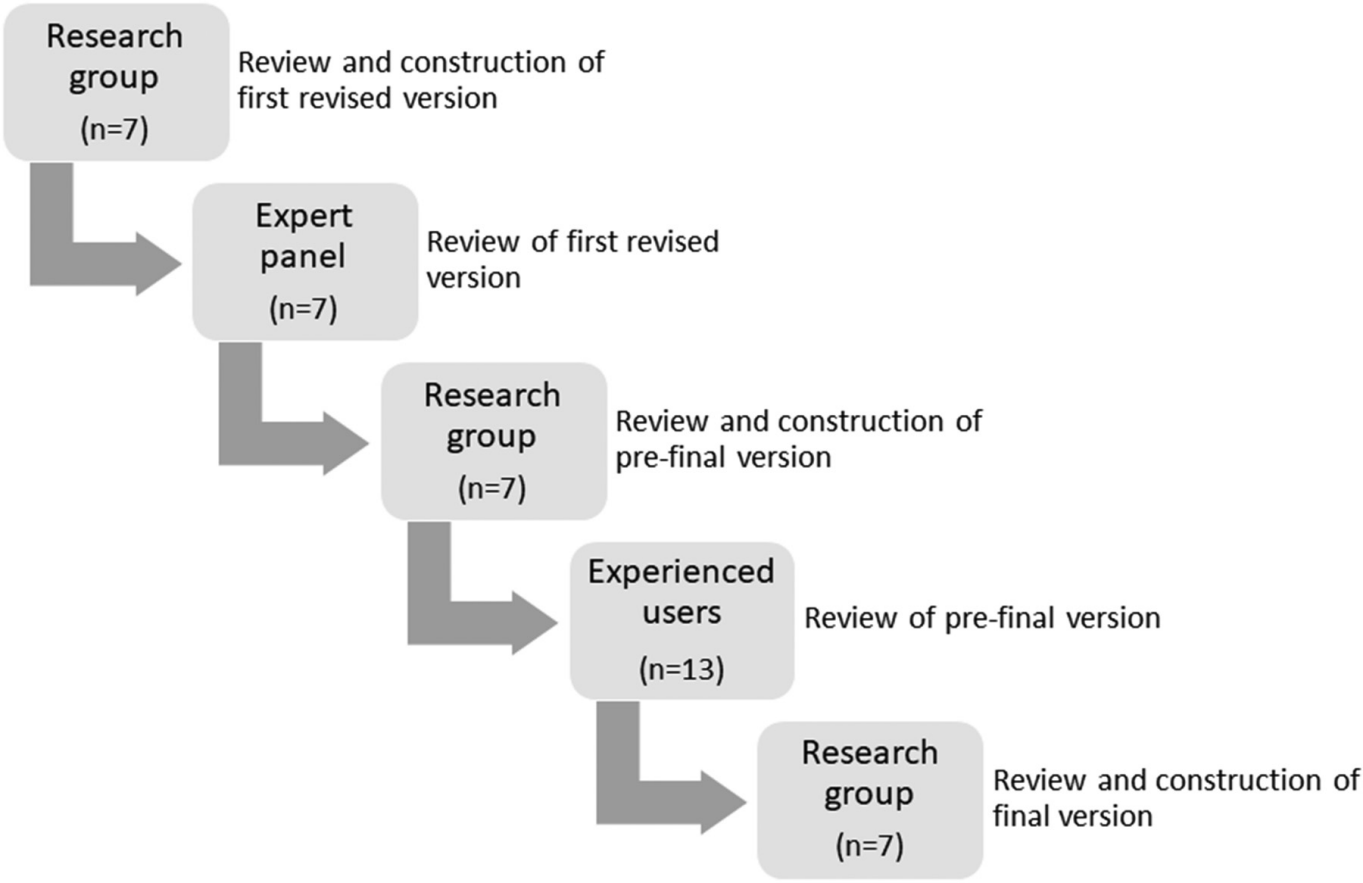
Graphic representation outlining the stages of the revision process for the DAP.

See Figure 1 for a graphical representation outlining the stages of the revision of the DAP.

Ethical approval for this study was obtained from the Swedish Ethical Review Authority, 2022-02587-01.

### Participants

#### Research group

The research group consisted of experts with good knowledge of the DAP, representing different areas within occupational healthcare and vocational rehabilitation: three physicians, an occupational therapist, and three registered physiotherapists specializing in ergonomics.

#### Expert panel

A panel of seven experts was recruited by a purposive sampling method.^31,33^ In the sampling, the intention was to recruit experts from diverse professions who have expertise in the areas of the DAP. They received an invitation to participate, together with written information about the study and the first version of the revised DAP by e-mail. They were also informed that they could withdraw from the study at any time for any reason. The participants accepted participation by answering the e-mail. The panel consisted of three physicians, two physiotherapists, one occupational therapist, and one clinical psychologist working in the field of occupational health. The mean age was 52 years, and the mean number of years of work experience was 24, ranging from 11 to 37 years.

#### Experienced users

The experienced users who participated in the Think Aloud interviews were also recruited by a purposive sampling method.^31^ The inclusion criterias were being a health professional, educated in the DAP, experienced in using the dialogue tool within occupational health services or healthcare settings. Eligible individuals were contacted by e-mail. They were asked if they were willing to participate in a Think Aloud interview and received written information about the aim of the study. The e-mail also included information about the right to withdraw from the study at any stage. Those who agreed to take part confirmed their participation by answering the e-mail and signing a written informed consent form. The interview group comprised five physiotherapists, of whom four were specialized in ergonomics, three occupational therapists, three occupational nurses, one occupational physician, and one human resources specialist in vocational rehabilitation. The mean age was 52 years, and the mean number of years of work experience was 28, ranging from 17 to 30 years, and the time of DAP-experience varied from 1 to 5 years.

### Data collection

#### Expert panel - questionnaire

The participants received a questionnaire containing the first revised version of the DAP. They were instructed to take a close look at the design, focusing on whether the items in the DAP were clear and easy to understand, if changes and additions should be made, or if any item should be removed. They were also asked to consider the relevance of the items in response to the working life and work environment of today. In the questionnaire, the experts could write their points of view with comments and suggestions linked to the respective items, proposing changes or improvements.

#### Experienced users - interviews

The Think Aloud interviews varied between 20 and 60 min, with four conducted face-to-face and nine held in digital meetings.^34,35^ All interviews were carried out by the first author and recorded. The interview guide consisted of the pre-final version of the revised DAP. Each session started with a brief explanation about how to perform the Think Aloud. Participants were instructed to systematically read aloud through the DAP-protocol, and item by item, think out loud what came to mind, not hesitating too long on each section, but instead moving forward at a steady pace. If hesitation occurred, the interviewer prompted the participant to continue reading and thinking aloud. The Think Aloud interview guide was piloted with the first participant, and no changes were deemed necessary. Therefore, the pilot interview was included in the study.

### Analysis

#### Research group

In reviewing previous research on the DAP, the suggested improvement measures were taken into account and discussed after each data collection. The researchers valued these suggestions together with their own notable propositions. The aim was to merge all the suggestions for improvements in a structured manner, for an optimal conclusion.

#### Expert panel - questionnaires

The questionnaires were compiled into a written report, in which answers from all participants regarding each item were brought together by the first author. Thereafter, similar wordings, comments, and additional suggestions were arranged in groups, making the compilation as rich and comprehensive as possible. The preliminary findings were then discussed within the research group. Based on these findings, the pre-final version of the revised DAP was designed.

#### Experienced users – interviews

Thematic analysis with a deductive approach was used to analyze the Think Aloud interviews. This process comprised several steps,^36^ beginning with the interviews first being transcribed verbatim and the material repeatedly read to get to know the material in-depth. All the interviews were thereafter analyzed by the first author using two predetermined concepts: (1) representativeness to the content domain and relevance to the construct of the DAP and (2) clarity and wording.^32^ The first concept refers to the extent to which the DAP reflects the characteristics of an individual's function and capability, related to the demands at work. It also pertains to the suitability for use in an existing context. The second concept concerns how the items are logical and easily understood, as well as how the content of the items is expressed. For each domain, the individual answers, item by item, were compiled to obtain a coherent and clear summary. To support the process of analyzing, memorandum notes were used during all steps. Thereafter, the research group discussed the findings related to the predetermined concepts, going back and forth between the original and the revised version of the DAP, exploring ways to consolidate until a consensus was reached. Moreover, additional suggestions and comments regarding the composition of the domains, considerations about interchanging the starting domain, and practical notes made by the participants were also compiled and synthesized. The research group made decisions on maintaining, modifying, or deleting an item through discussion. The last round of review produced the final version of the revised DAP, alongside a report with the content of the concepts and additional examples included.

## Results

### Research group

**Figure 2.**
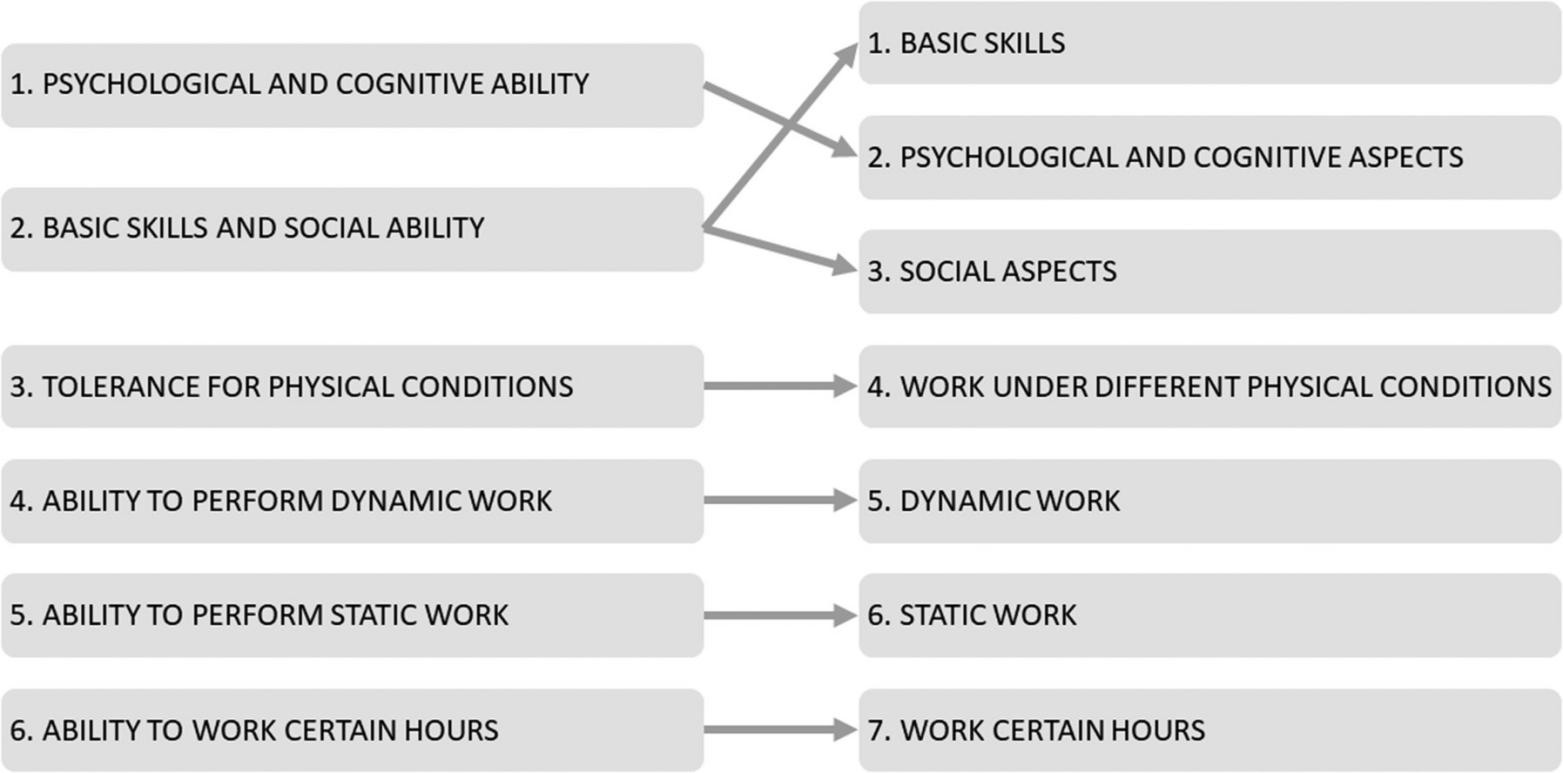
Overview of the restructured order sequence of the domains and revision of the names of the domains in the DAP.

The first step in the revision procedure was the research group's systematic review of the DAP, which resulted in initial changes. This review could be divided into three sections. First, the group restructured the order sequence of the domains, together with a revision of the domain names. In the original version, the DAP had six domains, starting with Psychological and Cognitive Ability, followed by Basic Skills and Social Ability. This order was changed, and the revised version starts with the domain on basic skills in order to have a more neutral focus at the beginning of the dialogue. This domain was also divided into two parts, separating Basic Skills from Social Ability, aiming at placing items about social aspects further ahead in the dialogue tool. Furthermore, in order to increase the stringency of formulations, some domains were reformulated (see Figure 2). These changes resulted in seven domains in the updated and revised first version of the DAP.

Second, the revision involved minor changes in the wording of instructions and the addition of instructions for conciseness in a number of items. This revision was intended to make the items within each domain as clear and easy to understand as possible.

The third section included adding an item to Basic Skills domain regarding the ability to count and removing an item from Dynamic Work domain that involved using a keyboard and mouse in computer work.

### Expert panel

The overall impression from the experts, after reviewing the first version of the revised DAP, was that the items in the DAP were clear and easy to understand. Accompanying this, the experts also wrote several qualitative comments, which could be divided into three main themes.

The first theme concerned how the DAP is performed in the clinical setting. The experts commented that it should be stated clearly in the introduction that the facilitator must be an educated healthcare or an occupational health professional. There was unanimous agreement on the importance of relevant background knowledge for the facilitator when conducting the DAP, highlighting its importance for the dialogue.

The second theme referred to the formulations regarding time and repetitions in some of the items in the DAP. Suggestions were made for reformulating or deleting these formulations. An expert described:

*The strength of the DAP can be seen, among other things, as that it is a relative instrument that puts ability in relation to demands. In some questions, there are definitions, formulated on the basis of absolute requirements: e.g., able to walk for more than an hour. In other questions, it is formulated based on a relative requirement: for example, being able to sit to the extent that the work requires. I think it would be good to determine an overall approach here.*

The third theme focused on suggestions for reformulating, improving, and combining heading and/or instruction texts in some of the items. Furthermore, regarding the fourth domain concerning the Ability to work dynamically, there were suggestions for improvement by merging items to eliminate overlap. With this convergence, the items were considered to become more distinct, thereby aiming to fully cover the intended ability.

Tables 1–6 show the major comments and suggestions regarding changes, improvements and revisions made by the research group, expert panel and experienced users in the domains and items of the DAP.

**Table 1.** Overview of the changes made in the domain basic skills and its link to the ICF.

BASIC SKILLS			
Original DAP	Revisions suggested by the research group, expert panel, and experienced users	New version of DAP	ICF Code
**Eyesight:**	**Research group:** Added instructions for conciseness	**To see:** *See and interpret visual impressions and be able to adapt the vision to the demands of the work tasks*	D110
	**Experienced users:** Changing the wording of the instructions for conciseness		
**Hearing:**	**Research group:** Added instructions for conciseness	**To hear:** *Hear, perceive, and interpret auditory impressions*	D115
	**Expert panel:** Item reformulated from: *To listen* to *To hear*		
**Speak:** *Be able to make oneself understood*		**To speak:** *Make oneself understood verbally*	D330
	**Research group:** Item added to domain: *To count*	**To count:** *Understand numbers and to calculate numbers*	D172
**To write:** *Be able to make oneself understood in writing*	**Research group:** Changing the wording of the instructions for conciseness	**To write:** *Make oneself understood in writing, have the ability to express oneself in writing, such as to spell correctly and use correct grammar*	D170
**To read:** *Be able to understand written information*	**Research group:** Changing the wording of the instructions for conciseness	**To read:** *Read and comprehend written information*	D166

**Table 2.** Overview of the changes made in the domain psychological and cognitive aspects and its link to the ICF.

PSYCHOLOGICAL AND COGNITIVE ASPECTS			
Original DAP	Revisions suggested by the research group, expert panel, and experienced users	New version of DAP	ICF Code
**Divided attention:** *Be able to pay attention simultaneously to several sources of information for at least 30 min*	**Research group:** Time measurement was removed	**Divided attention:** *Being aware of several sources of information, tasks at the same time*	D1601
**Memory:** *Be able to remember relevant information when it is needed*	**Research group:** Added instructions for conciseness	**Memory:** *Remember relevant information when it is needed and learn new elements*	B144
**Self-awareness:** *Be able to realize their own abilities and limitations*		**Self-awareness:** *Recognize one's own abilities and limitations*	B180
**Act goal-oriented:** *Be able to plan their own work in order to achieve set goals*		**Act goal-oriented:** *Plan your own work to achieve set goals*	D177
**Act independently:** *Be able to carry out tasks independently*	**Expert panel:** Changing the wording of the instructions for conciseness	**Act independently:** *Plan, make choices between options, and perform tasks independently*	B164
**Tempo:** *Be able to perform tasks quickly*	**Research group:** Changing the wording of the instructions for conciseness	**Tempo:** *Perform tasks quickly and adapt the pace based on what the tasks require*	D230

**Table 3.** Overview of the changes made in the domain social aspects and its link to the ICF.

SOCIAL ASPECTS			
Original DAP	Revisions suggested by the research group, expert panel, and experienced users	New version of DAP	ICF Code
**Ability to express one's own feelings:** *Be able to express emotions in words and actions*	**Expert panel:** Change of wording of heading and instructions for conciseness	**Expressing your own feelings:** *Expressing and adapting emotions in words and actions*	D720
	**Experienced users:** Changing the wording of the instructions for conciseness		
**Handle conflicts:** *Be able to handle a conflict face-to-face*	**Research group:** Changing the wording of the instructions for conciseness	**Handle conflicts:** *Dealing with a conflict situation*	D7202
**Collaboration with others:** *Be able to perform tasks in collaboration*	**Research group:** Changing the wording of the instructions for conciseness	**Collaboration with others:** *Performing tasks together with others, creating and maintaining specific relationships in different contexts*	D710 D740
	**Experienced users:** Changing the wording of the instructions for conciseness		
**Transport:** *Be able to drive a car, cycle, or independently use public transport*	**Experienced users:** Original item #5 divided into items #5 and #6 to cover the entire intended area	**Transport:** *Driving, cycling, or using public transport to and from work*	D470
		**Transport:** *Driving, cycling, or using public transport during working hours*	D470

**Table 4.** Overview of the changes made in the domain work under different physical conditions and its link to the ICF.

WORK UNDER DIFFERENT PHYSICAL CONDITIONS			
Original DAP	Revisions suggested by the research group, expert panel, and experienced users	New version of DAP	ICF Code
**Cold:** *Be able to tolerate severe cold temperatures*		**Cold:** *Working in extreme cold temperatures*	D5700
**Draft:** *Be able to tolerate draughts and wind*		**Draft:** *Working in draughts and winds*	D5700
**Skin contact:** *Be able to tolerate intensive contact with solid and/or liquid substances*	**Expert panel:** Changing the wording of the instructions for conciseness	**Skin contact:** *Working with regular contact with solid and/or liquid substances*	D5700
	**Experienced users:** Item reformulated from *Intensive skin contact → Regular skin contact*		
**Protective measures:** *Be able to tolerate the use of personal or other protective equipment*		**Protective measures:** *Working with personal or other protective equipment*	D5700
**Dust, smoke, gas, and mist:** *Be able to tolerate work in an environment with dust, fumes, gases, and/or moisture*	**Experienced users:** Changing the wording of the heading and instructions for conciseness	**Dust, smoke, gas, and moisture:** *Working in environments with dust, fumes, gas, humidity*	D5702
**Noise:** *Be able to tolerate working in a noisy environment*		**Noise:** *Working in a noisy environment*	D5700
**Vibration:** *Be able to tolerate work with vibrating tools or in an environment with whole body vibration*		**Vibration:** *Working with vibrating tools or in an environment with whole-body vibration*	D5702

**Table 5.** Overview of the changes made in the domain dynamic work and its link to the ICF.

DYNAMIC WORK			
Original DAP	Revisions suggested by the research group, expert panel, and experienced users	New version of DAP	ICF Code
		**Touch/touch:** *Feel surfaces and their texture or quality*	D265
**Stretching the arm at work:** *Even several times a minute and for a large part of the working day* **Handling of light objects (not more than 1 kg):** *Even several times a minute and for a large part of the working day*	**Expert panel:** Items #4 + 5 in the original version were merged. Since the items related to each other, they could be clarified and simplified	**Able to stretch and bend the arm repeatedly:** *Perform tasks that require stretching and/or bending the arm repeatedly*	D430
**Forward bend:** *Be able to pick up something from the floor, even several times per minute and for a large part of the working day* **Twisting of the body:** *Be able to turn the body at least 45 degrees, even several times per minute and for a large part of the working day*	**Expert panel:** Items #6 + 7 in the original version were merged. Since the items related to each other, they could be clarified and simplified	**Bending and twisting the body:** *Carry out tasks that require bending and/or twisting the body, picking up something from the floor or leaning forward without support*	D450
**Drag and slide:** *Be able to pull and push, even several times a minute and for a large part of the working day*	**Expert panel:** Changing the wording of the instructions for conciseness	**Push and pull:** *Pushing and pulling objects*	D4550
	**Experienced users:** Change of wording of the heading		
**Carrying and handling heavy objects (approx. 15 kg):** *Occasionally, even several times per hour and for a large part of the working day*	**Expert panel:** Changing the wording of the instructions for conciseness	**Carrying and handling heavy objects:** *Carrying and handling heavy objects (approx. 15 kg)*	D430
**Head movements:** *Be able to move your head in all directions*		**Head movements:** *Able to move your head in all directions*	D4108
**Walking work:** *Be able to walk, about 1 h at a time and also for a large part of the working day*	**Experienced users:** Changing the wording of the instructions for conciseness	**Walking work:** *Walk during the workday*	D450
**Stairway:** *Be able to go up and down stairs without stopping*	**Experienced users:** Changing the wording of the instructions for conciseness	**Stairway:** *Going up and down stairs*	D451
**Climbing:** *Be able to climb up and down a ladder without stopping*	**Experienced users:** Changing the wording of the instructions for conciseness	**Climbing:** *Climbing up and down a ladder or other height*	D455
**Kneeling/squatting:** *Be able to reach the ground/floor with your hands when kneeling or squatting*	**Expert panel:** Changing the wording of the heading and instructions for conciseness	**Changing body position:** *Sitting down and getting up from kneeling or squatting*	D4101 D4102

**Table 6.** Overview of the changes made in the domain static work and its link to the ICF.

STATIC WORK			
Original DAP	Revisions suggested by the research group, expert panel, and experienced users	New version of DAP	ICF Code
**Standing posture:** *Be able to stand without interruption (approx. 1 h) and also throughout the entire working day*	**Expert panel:** Changing the wording of the instructions for conciseness	**Standing posture:** *Working in a standing position*	D4153
**Work in a kneeling position or squatting** *: Be able to work in this position (about 5 min at a time) and repeatedly during the working day*		**Kneeling or squatting work:** *Working in a kneeling or squatting position*	D4151 D4152
**Working in a bent or twisted position** *: Be able to work in this position (about 5 min at a time) and repeatedly during the working day*		**Forward bend or twisted working position:** *Working bent forward or in a twisted position*	D4158
**Work with the arms above shoulder height:** *Be able to work in this position (about 5 min at a time) and repeatedly during the working day*		**Work with the arms above shoulder height:** *Working with your arms above shoulder height*	D4158
**Keep your head in a fixed position while working:** *Be able to keep your head in the same position even during a large part of the working day (e.g., look up/to the side)*		**Keep your head in a fixed position while working:** *Keeping your head in a fixed position*	D4155

### Experienced users

The results of the Think Aloud interviews are presented domain by domain, according to the two concepts that were covered in the interview guide: 1) representativeness and relevance, and 2) clarity and wording.

#### Basic skills

*Representativeness and relevance.* The characteristics of the items included were considered representative, effectively covering the content. The DAP-users expressed that information regarding basic skills encompassed more aspects than first anticipated, providing valuable dimensions. One participant remarked, “*So that they* [the issues] *have been more complex than I first thought, actually and it also goes deeper, or became, when I've used it.”* The basic skills were acknowledged to have a significant impact on the RTW process, thus highlighting their relevance for the DAP in assessing employees’ functions and their demands at work.

*Clarity and wording*. Overall, the items were found to be clear, and the language simple. However, the DAP-users observed that some items had a broad approach, which could pose a challenge for both employers and employees to grasp. This made the role as a facilitator extra important in leading the DAP dialogue. Nevertheless, they expressed that they understood the items and could thus effectively guide and facilitate the process. An overview of changes made in this domain based on the findings is shown in Table 1.

#### Psychological and cognitive aspects

*Representativeness and relevance.* In this domain, the DAP-users pointed out the importance of each one of the items being discussed and found the content to be multifaceted. During discussions on the balance between demands and function, some DAP users discussed whether it would be beneficial to have time limits to relate to. One user stated, “*It would be good to have - within that time, with some time aspect or time indication. But that's what I've also thought when I've used the DAP, for how long can you expect* [to do this].” However, the overall conclusion, when also including the comments from the expert panel, was not to include such time limits.

*Clarity and wording.* The participants found the items in this domain to be significantly clear, thus easy to carry out in the DAP dialogue. However, some DAP users mentioned that psychological and cognitive terms could sometimes be difficult to explain to both the employee and employer. They emphasized the importance of carefully considering the individual employee, employer, and the specific context. One user expressed, “*I've sat with this and thought about* [the question]*, but I understand that it's just who you sit with and then it's how you use the word.”* An overview of changes made in this domain based on the findings is shown in Table 2.

#### Social aspects

*Representativeness and relevance.* The items in this domain were, for the most part, considered representative by the DAP users. They emphasized the importance of being able to discuss social aspects and the employee's functional ability in relation to the demands of their work. *“I think it absolutely shows both what demands are made by the employer and also for the individual, so it emerges and leads to good discussions*.” The items, all together, created a genuine platform for discussing these issues in relation to the work tasks.

*Clarity and wording.* The items were regarded as clear and straightforward. However, even though the items were considered clearly formulated, some DAP users described challenges with the employee and employer in answering and discussing the items due to their complexity. One user commented, “*Obvious question but* [the topic] *can be difficult to talk about.”* Despite this, there was a consensus that the wording in this domain was not complicated, and the explanations for each item made it easy to use. Some remarks were made that if misinterpretation occurred, it welcomed the opportunity for a constructive discussion. One user mentioned, “*For example, I know about* [items where] *there is usually a bit of a question mark* [then], *there will be a discussion then, about what it is like, and that is good.”* An overview of changes made in this domain based on the findings is shown in Table 3.

#### Work under different physical conditions

*Representativeness and relevance*. In this domain, the DAP users agreed that the DAP covered significant dimensions present in the physical work environment. However, it was pointed out that it also meant that some content was not universal and therefore did not apply to all employees. On the other hand, this was considered an asset, contributing to interesting viewpoints on different work environments and their pros and cons.

An explicit observation from some DAP users was not to rush through or skip any item in this domain. This was to ensure that nothing in the physical work environment that could be of importance regarding the employee's functional ability, linked to the demands of the work, and thus influencing work ability, was overlooked. One user commented:

*It's also something like that, I took that DAP-course quite a while ago. And I use it often. And at some point, I've thought that, for example, if I talk to a care assistant in home care, it feels a little unnecessary. But you have to ask all the questions because then this woman said that it is actually a relevant question because I can't shower my patients, because then I get angina. Yes, you see…so you have to ask everything.*

*Clarity and wording*. In this domain, the items appeared clear and distinct. Here, it also emerged that the DAP users could experience that the more specific an item, the more important it was for them to have a grasp of the wording and to have a good explanation for what was meant, to lead the employee and employer on to the right track. An overview of changes made in this domain based on the findings is shown in Table 4.

#### Dynamic work

*Representativeness and relevance*. The content of the domain was considered representative, with items referring to many important aspects of dynamic work. Some items had been merged in the pre-final revised version of the DAP, which was reflected on by the DAP users*.* It all came down to the majority expressing that the merging was beneficial, as it could lead the dialogue into areas that had not previously been discussed. One user stated, *“Because then it was more that* [the questions] *were more precise, so that they were limiting. Here are more open questions.”*

*Clarity and wording*. The wording was found to be clear and the language simple and obvious in this domain. Users stated: *“This is better, this is clearer.”* Overall, it was expressed that the subheadings and instructions facilitated the employee and employer to really look closely at the task at hand, thus enhancing the dialogue. An overview of changes made in this domain based on the findings is shown in Table 5.

#### Static work

*Representativeness and relevance.* It was emphasized that consistency was very important in discussing the kind of workload. Sticking to the requested domain required some extra thought and was therefore important to clarify from the beginning, together with the employee and employer. One user highlighted:

*Exactly, if you think statically, that this is under the heading static, then I think like this […] you have to differentiate between it, because there you often go wrong, dynamically or staticall*y.

Discussion of static working positions and the employee's functional ability was considered important in relation to the work demands.

*Clarity and wording*. In this domain as well, the items were found to be clear and evident. Their formulations were precise and could not be misinterpreted, with the subheadings and instructions providing support. An overview of changes made in this domain based on the findings is shown in Table 6.

#### Work certain hours

*Representativeness and relevance.* The DAP users pointed out that the three questions in the domain offered an interesting approach when discussing the ability and function related to time at work. A comment that was repeated by several was the need to delve deeper into each item to fully grasp the situation. Several users stated, “*because there is a lot to gain from the questions in this domain if you try to reach beyond certain hours.”* This way of considering working hours allows the domain to be more universal and applicable to any kind of working situation or profession. Talking about working hours could spark a good dialogue about whether the employee will return gradually or full-time. On the other hand, there were also voices raised about the items shifting the attention away from the actual time spent at work to the ability to get work done during those hours. Some users expressed, “*I mean, there are people being able to be at work for 8 h, but they are getting nothing done.”*

*Clarity and wording.* The majority of users declared the items to be clear and distinct. No changes were made regarding formulations; however, Table 7 presents the items in the first version and the final version of the revised DAP.

**Table 7.** Overview of the domain work certain hours and its link to the ICF.

WORK CERTAIN HOURS			
Original DAP	Revisions suggested by the research group, expert panel, and experienced users	New version of DAP	ICF Code
**Times of the day:** *Work at all hours of the day, even at night*		**Times of the day:** *Work at all hours of the day, even at night*	D8502
**Hours per day:** *Work about 8 h per shift*		**Hours per day:** *Work about 8 h per shift*	D8502
**Hours per week:** *Work about 40 h per week*		**Hours per week:** *Work about 40 h per week*	D8502

#### Additional comments and suggestions

In the first revision, the research group divided the domain of Basic Skills and Social Abilities into two separate domains. The starting domain was also altered by allowing Basic Skills to initiate the DAP instead of Psychological and Cognitive Aspects. This change was received very positively by most of the DAP users. One user claimed, “*The basic and the social aspects were together earlier; they are the ones you have split. Yes, I think it is good and I think it will be clearer to divide them into two.”* This change was considered to make the content clearer. Another user stated, “*To have the social aspects as a separate domain, that it clarifies what areas we are talking about, what focus I should have and think about when I answer these questions. I think that also makes it clearer.”* It also provided an opportunity to address the capabilities and functions that perhaps could be more challenging for the employee and employer to grasp and discuss. One user remarked, “*I think it's easier to, when you get a little bit warmed up, yes, I think that's a good choice. That being said, overall, an improvement, clearly, and I think it was good restructuring it.”*

It was further commented that the generic construction of the DAP was an asset when focusing on the individual's functional capacity in the DAP dialogue. One user commented:

*It is a challenge to take a large person and a small person who is normally functioning, and they will have very different power […] like yes, we are all functioning, but we will cope very differently, and so how much can the employer demand? DAP can be a help here.*

In these situations, it was suggested that if the DAP user had a pre-understanding about the demands in the particular working situation, it could facilitate the dialogue in many ways. One DAP user said:

*I always usually, I have learned, request the job description and together with the customer and the manager say that: like this, this is what I have learned that you do as a groundskeeper, preschool teacher, or something like that, that you have as a basis.*

## Discussion

The study aimed to update and revise the content of the ICF-based instrument Demand and Ability Protocol (DAP) as it has not been revised since its introduction in Sweden, nor has the content validity been tested. With the consensus of a research group, an expert panel, and health professionals experienced in using the DAP, the content validity of the dialogue tool was assessed, and the domains and items were updated. This paved the way for a thorough procedure that resulted in the revised version of the DAP. The findings revealed that the DAP is considered a dialogue tool with good content validity, thus easy to understand with clear wordings and structure.

A significant change made in the DAP was the alteration of order of the domains and the division of two domains. By initiating the dialogue with basic and more neutral items, then moving forward to areas that could be sensitive or difficult to discuss, a confident relationship can arise in the dialogue aiming for coherency and problem-solving.^37^ This is an important change being made as it is known that creating trusting and successful communication enables people involved to feel safe and acknowledged within the structure of the conversation.^14,38,39^ Furthermore, it is the facilitator's role in the conversation to ensure that these requirements are fulfilled.^14,40^ The changed structure of the revised DAP can influence the implementation, thus enhancing the opportunities of cooperation in a three-party meeting where the dialogue tool is being used. To facilitate the cooperation between the employee and various stakeholders is important early on, together with workplace interventions, since such efforts have been shown to have a significant impact on work ability and RTW.^13,15,41–43^ Besides being a dialogue tool, the DAP may also be an instrument to assess and explore the objectives of occupational rehabilitation and sustainable work ability. Our study suggests that the DAP is relevant in such situations and can be a useful tool for facilitating those factors.

Furthermore, since the DAP is a generic tool not specified for any medical diagnoses or conditions, nor any specific phase of rehabilitation it is possible for an employer to be involved in the three-party meetings using the same structure for employees with various diagnoses or requirements. This might be beneficial as the structure becomes familiar and is particularly unique for the DAP as the previously mentioned dialogue tools,^16–18^ consisting of various interview models, are intended for use in RTW-processes where the employee has been diagnosed with common mental disorders, stress-induced exhaustion disorder, or musculoskeletal disorders. The DAP can thus be considered as an optional alternative when an employee's ability does not match the demands at work, regardless of diagnosis or whether they are on sick leave or not. Using the same dialogue tool for various employees might strengthen the recognizability and thus promote the usability of the tool.

In summary, this study outlines the process of revising the DAP as a relevant dialogue tool to support employees, employers, and facilitators in health and occupational healthcare when discussing work ability. The revision and validation of the DAP have clarified the theoretical concepts and the link to the ICF, which creates the basis for the content of the dialogue tool. It also provides insights into the use of DAP in a clinical setting, even though that has not been the primary focus of this study. However, it should be noted that the DAP is a dialogue tool that is facilitated by a representative with a medical background and is not a self-report measure. This generates other demands on the formulations, with the most important being that the facilitator understands it well enough to describe and explain the domains to the employer and the employee. This study has contributed to increased understanding of each domain, and the results indicate that the tool is easy to comprehend and use for facilitators using the DAP.

### Strengths and limitations

Methodologically, this study was carried out using qualitative methods. There are various procedures available to assess content validity and no single method is considered the most effective to choose.^44^ Using quantitative methods and statistical measures of data can offer objective decisions by calculating content validity indices.^44,45^ However, the benefit of using a qualitative approach in the content validity procedure lies in the possibility of gaining a deeper understanding of the participants’ experiences.^46^

The results of the study enforced that the qualitative methods were valuable, as the panel of the proficient experts could describe their thoughts about the DAP in-depth. They could identify items, suggest changes and improvements in relation to the work situation and work environment of today. If we had chosen only quantitative measures, these clarifications and additional values would not have been provided.^47^ However, it is important to notice that the findings from a qualitative study cannot be generalizable. The transferability of the study has been strengthened by a thorough description of the context, participants and the design, to make it possible to recapture an assessment of similarity.^48,49^

Furthermore, the qualitative method of Think Aloud interviews offered profound insights from the DAP users about their perceptions, beliefs, challenges, and factors contributing to success regarding the relevance of the questions used in the dialogue tool.^47^ The results of this study suggest that the interviews with the DAP users provided additional important information in helping to increase relevance by identifying redundant and irrelevant items. It also provided valuable knowledge on how headings and instructions could be clarified. Further research is currently conducted by the research group focusing on the usability of the DAP in clinical settings.

In this study it is important to exercise caution regarding the sample size and distribution when interpreting the findings of the study.^50^ If the study had been carried out with more participants, the results might have been different. Although the sample size was not large, significant consensus was found in the compilation of the experts’ comments and in the analysis of the interviews. In addition, the experts and the DAP users were experienced, interested, and willing to contribute to the development of the tool. In all, this suggests an effective degree of redundancy.^51^ Furthermore, it was desirable to have as many men as women in the panel and the Think Aloud interviews. However, this was not achieved and may lead to gender bias. Nevertheless, it also reflects and is representative of how gender distribution is present in health and occupational care. We do have a variation concerning professions, however, which is a strength of the study. Potentially, the educational background might influence the understanding of the domains and thus be easier, as opposed to harder to understand, depending on the profession of the facilitator. Based on the varied sample, this pitfall is limited and provides a neutralized picture of how the formulations and relevance of the DAP are experienced.

## Conclusion

The DAP was revised and assessed for content validity using qualitative methods, incorporating multidisciplinary consensus from researchers, experts, and DAP users. Several significant changes were made in the domains to enhance clarity and conciseness. Some formulations were identified and changed, even though the questions were mostly perceived as clear and relevant. The DAP can, thus, be considered a dialogue tool with good content validity. The revision and validation process clarified the theoretical concepts and strengthened the link to the ICF, establishing the basis for the content of the dialogue tool.
